# Fourier Ambiguity Validation for Carrier-Phase GNSS

**DOI:** 10.3390/s26072201

**Published:** 2026-04-02

**Authors:** Peter J. G. Teunissen

**Affiliations:** 1 Department of Geoscience and Remote Sensing, Delft University of Technology, Stevinweg 1, 2628 CN Delft, The Netherlands; p.j.g.teunissen@tudelft.nl; 2 Department of Land Surveying and Geo-Infomatics, PolyU, Hong Kong, China; 3 Department of Infrastructure, University of Melbourne, Melbourne 3010, Australia; 4 GNSS Research Centre, Curtin University of Technology, Perth 6102, Australia

**Keywords:** Integer-Aperture (IA) estimation, Global Navigation Satellite Systems (GNSS), fourier ambiguity validation, mixed-integer model, Integer-Aperture Bootstrapping (IAB), Hybrid Spatial-Frequency IAB

## Abstract

Carrier-phase ambiguity validation is essential to ensure the reliability of integer ambiguity resolution in high-precision GNSS positioning. Although integer equivariant (IE) estimators provide optimal integer candidates within their class, noise and model limitations may lead to incorrect fixing. Validation procedures are therefore crucial for safeguarding the transition from float to fixed solutions, particularly in high-precision and safety-critical applications. In this contribution we introduce the concept of Fourier ambiguity validation and show how it is rooted in the principles of integer aperture (IA) estimation and its periodic representation. Unlike classical integer estimators that always return an integer solution, IA estimators introduce adjustable acceptance regions in the float ambiguity domain and fix ambiguities only when sufficient statistical evidence is present. As a result we present a general Fourier representation of IA estimators and provide an analytical description of the probabilistic properties of integer-aperture bootstrapping. We also present a hybrid description and show how the spatial and frequency representations can be mixed so as to do justice to the practical situation when carrier-phase ambiguities have a wide range of varying precision.

## 1. Introduction

Efficient carrier-phase ambiguity resolution is fundamental to achieving high-precision GNSS positioning. Once the integer ambiguities of the carrier-phase observations are correctly fixed, the carrier-phase measurements effectively act as highly precise pseudorange data, enabling centimeter- to millimeter-level positioning and navigation accuracy. The success of this process, however, depends not only on resolving the ambiguities as integers but also on ensuring that the accepted integer solution is statistically reliable. Without proper validation, the precision potential of carrier-phase data cannot be safely exploited in demanding real-time or safety-critical environments [1,2,3,4,5].

Statistical ambiguity validation addresses the question of whether a resolved integer ambiguity vector can be accepted as correct with controlled risk. Although integer estimators may provide a candidate integer solution, the presence of measurement noise and model misspecification can lead to incorrect fixing. An erroneously accepted ambiguity solution introduces biases directly into the positioning results and may degrade accuracy by orders of magnitude. A proper validation strategy must therefore balance reliability and efficiency: overly permissive criteria increase the probability of incorrect fixes, whereas overly conservative strategies reduce fixing rates and delay convergence to high-precision solutions. This need for a principled acceptance mechanism leads to the framework of integer aperture (IA) estimation as was introduced by the author in [6].

The class of IA estimators provides the theoretical foundation for carrier-phase ambiguity validation by introducing adjustable acceptance regions in the float ambiguity domain. Situated between the classes of integer estimators and integer-equivariant (IE) estimators, IA estimators allow the decision to fix or to withhold fixing to be governed by statistical evidence. They have therefore found widespread use in carrier-phase GNSS, with typical examples being the ratio test, the difference test, and the projector test [7,8,9]. A review and evaluation of these IA estimators, together with modifications, can be found in, for instance, the studies [10,11,12].

The novelty of the current contribution is that we reveal and exploit the $Z^{n}$-periodic structure of IA estimators, which enables us to develop new, frequency-domain representations for all members of this class. By recognizing that ambiguity residual functions are periodic over the integer grid, we develop and establish a general Fourier representation in Section 2, thereby complementing the conventional spatial formulation. By revealing the harmonic structure underlying ambiguity validation, we are able to show for the first time how a mixing of the spatial and frequency representations can lead to computational advantages in the practical situation when carrier-phase ambiguities have a wide range of varying precision.

On this basis, Section 3 focuses on integer-aperture bootstrapping (IAB), one of the most practical and computationally efficient members of the IA-class. The IAB estimator, introduced by the author in [13], has been used for various high-integrity carrier-phase applications [14,15,16,17,18]. IAB is attractive not only because of its simplicity but also because its probabilistic performance can be described analytically. We derive explicit analytical expressions for its success, failure, and undecided probabilities and show how these are governed by the spectrum of sequential conditional ambiguity variances. A generalized version of Poisson’s summation formula is then introduced to obtain an alternative analytical representation of these performance measures in the frequency domain, thereby linking probabilistic behavior directly to spectral characteristics.

Three complementary formulations—spatial, frequency, and hybrid—are finally brought together in Section 4. While the spatial representation provides intuitive geometric insight and the frequency representation exposes the harmonic structure of validation, neither alone is sufficient for modern multi-GNSS practice, where ambiguity vectors often contain components with widely differing precision levels. The proposed analytical hybrid formulation therefore combines spatial and spectral elements in a unified framework, offering the flexibility required to accommodate heterogeneous ambiguity configurations and to support reliable ambiguity validation in realistic high-precision applications.

The following notation is used throughout. E(.) stands for the mathematical expectation operator and $N_{m}\left(\mu ,Q\right)$ denotes an *m*-dimensional, normally distributed random vector, with mean (expectation) μ and variance matrix (dispersion) *Q*. The Best Linear Unbiased Estimator (BLUE) of a parameter vector *x* is denoted as $\hat{x}$ and its IA estimator as $\overset{ˇ}{x}$. The probability density function (PDF) of random vector $\hat{x}$ is denoted as $f_{\hat{x}}\left(x\right)$.$R^{p}$ and $Z^{p}$ denote the *p*-dimensional spaces of real- and integer numbers, respectively, and C the set of complex numbers. |M| denotes the determinant of matrix *M*, ⌊x⌉ denotes rounding scalar *x* to the nearest integer, and $\prod_{i=1}^{n}a_{i}=a_{1}\times \ldots \times a_{n}$. The $Q_{yy}$-weighted squared norm is denoted as ${\left||.|\right|}_{Q_{yy}}^{2}={\left(.\right)}^{T}Q_{yy}^{-1}\left(.\right)$.

## 2. Integer-Aperture (IA) Estimation

### 2.1. GNSS Ambiguity Resolution

To formulate our GNSS model of observation equations, we assume the vector of observables to be distributed as $y∼N_{m}\left(E\left(y\right),Q_{yy}\right)$, with its mean parametrized as [4,5]

(1) $$E\left(y\right)=Aa+Bb,\;a\in Z^{n},b\in R^{p}$$

in which (A,B) is a given m×(n+p) matrix of full rank. The *m*-vector *y* contains the carrier-phase and pseudorange observables, the *n*-vector *a* the integer-estimable ambiguities, and the real-valued *p*-vector *b* the remaining unknown parameters, such as baseline components (coordinates), clock biases, instrumental delays and possibly atmospheric delay parameters (troposphere, ionosphere). The matrix (A,B) contains the relative receiver-satellite geometry and the wavelengths of the carrier-phase data. Below, *a* and *b* will refer to the ambiguity vector and the baseline vector, respectively.

If we denote the best linear unbiased estimators (BLUEs) of *a* and *b* as $\hat{a}$ and $\hat{b}$, respectively, then the integer-ambiguity resolved solutions of *a* and *b* are given as

(2) $$\overset{ˇ}{a}=I\left(\hat{a}\right)\;\;\mathrm{and}\;\;\overset{ˇ}{b}=\hat{b}-Q_{\hat{b}\hat{a}}Q_{\hat{a}\hat{a}}^{-1}\left(\hat{a}-\overset{ˇ}{a}\right)$$

in which $I:R^{n}\mapsto R^{n}$ is an *integer-equivariant* mapping [19], $Q_{\hat{a}\hat{a}}$ the variance matrix of $\hat{a}$ and $Q_{\hat{b}\hat{a}}$ the covariance matrix of $\hat{b}$ and $\hat{a}$. The mapping from $\hat{a}$ to $\overset{ˇ}{a}$, and thus the choice of I(.), is referred to as integer ambiguity resolution (IAR). It is here where a choice for the integer-equivariant mapping I(.) needs to be made. For this choice, three different classes of ambiguity estimators can be discriminated: the class of integer (I) estimators, the class of integer-aperture (IA) estimators and the class of integer-equivariant (IE) estimators. The three classes have the following natural ordering,

(3) I⊂IA⊂IE

IE estimators form the largest class, while I estimators form the smallest. As our focus is on *ambiguity validation*, we will work in this contribution with the IA class. Corresponding theory for the IE class can be found in [20].

### 2.2. Integer and Integer-Aperture Estimation

Since the IA-class is a relaxed version of the I-class, we first briefly describe the latter. In case of the I-class, we have $I:R^{n}\mapsto Z^{n}$, such that the subsets $P_{z}=\left\{x\in R^{n}|\;z=I\left(x\right)\right\}\subset R^{n}$ form a *z*-translational invariant partitioning of $R^{n}$, i.e., $\cup_{z\in Z^{n}}P_{z}=R^{n}$, $P_{u}\cap P_{v}=\emptyset$ for u≠v, and $P_{z}=P_{0}+z$, $\forall z\in Z^{n}$. The subsets $P_{z}$ are called *pull-in regions* as any $\hat{a}\in P_{z}$ will be pulled to *z* by the integer mapping I. Popular I estimators are integer rounding (IR), integer bootstrapping (IB) and integer least-squares (ILS); see e.g., [21,22].

The IA-class is a relaxed version of the I-class and was introduced by the author so as to be able to combine ambiguity estimation with ambiguity validation; see e.g., [13,23]. It relaxes I estimation in the sense that instead of mapping the whole of $R^{n}$ to $Z^{n}$, IA estimators map only a *z*-translational invariant subset $\Omega \subset R^{n}$ to the space of integers, while acting as an identity map on its complement $R^{n}\setminus \Omega$. Thus for Ω=Ω+z, we now have $I\left(\hat{a}\right)=\hat{a}$ if $\hat{a}\in R^{n}\setminus \Omega$ and $I:\Omega \mapsto Z^{n}$, such that the subsets $\Omega_{z}=\left\{x\in \Omega |\;z=I\left(x\right)\right\}$ form a *z*-translational invariant partitioning of Ω. If we let $\omega_{z}\left(x\right)$ be the indicator function of $\Omega_{z}$, i.e., $\omega_{z}\left(x\right)=1$ for $x\in \Omega_{z}$ and $\omega_{z}\left(x\right)=0$ otherwise, then any IA estimator can be written as

(4) $$\overset{ˇ}{a}=\hat{a}+\sum_{z\in Z^{n}}\left(z-\hat{a}\right)\omega_{z}\left(\hat{a}\right)$$

**Figure 1:**
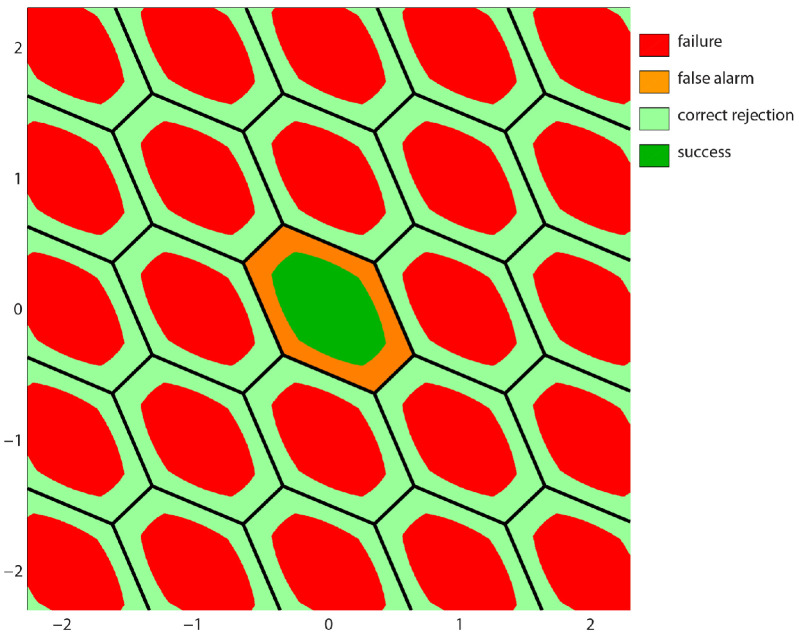
Two dimensional IA pull-in regions $\Omega_{z}$ as subsets of hexagonal pull-in regions $P_{z}$: green for correct integer estimation ($\hat{a}\in \Omega_{a}$); red for incorrect integer estimation ($\hat{a}\in \cup_{z\in Z^{n}}\Omega_{z}\setminus \Omega_{a}$); orange for false alarm ($\hat{a}\in P_{a}\setminus \Omega_{a}$); light green for correct detection ($\hat{a}\in \left(R^{2}\setminus \Omega \right)\setminus \left(P_{a}\setminus \Omega_{a}\right)$).

Note that the *z*-translational invariance $\Omega_{z}=\Omega_{0}+z$ implies $\omega_{z}\left(x\right)=\omega_{0}\left(x-z\right)$. Thus any IA estimator is uniquely characterized by $\omega_{0}\left(x\right)$ and thus by $\Omega_{0}$. This shows that the subset $\Omega_{0}$ plays the same role for IA estimators as $P_{0}$ does for I estimators. By changing the size and shape of $\Omega_{0}$ one changes the outcome of the IA estimator. The subset $\Omega_{0}$ can therefore be seen as an adjustable pull-in region with two limiting cases. The limiting case in which $\Omega_{0}$ is empty and the limiting case when $\Omega_{0}$ equals $P_{0}$. In the first case, the IA estimator becomes identical to the float solution $\hat{a}$ and in the second case the IA estimator becomes identical to an I estimator. The subset $\Omega_{0}$ therefore determines the *aperture* of the pull-in region. Figure 1 shows a 2D example of integer-aperture pull-in regions. The green and red regions correspond to correct and incorrect integer outcomes, while the orange and light green areas correspond to real-valued IA-outcomes (orange for false alarm and light green for correct detection).

### 2.3. Fourier Representation of I and IA Estimators

By noting that the ambiguity residual vector $\hat{a}-\overset{ˇ}{a}=\sum_{z\in Z^{n}}\left(\hat{a}-z\right)\omega_{z}\left(\hat{a}\right)$ is a $Z^{n}$-periodic function of the float solution $\hat{a}$, we may now develop the theory of GNSS ambiguity validation further by means of results known from multivariate Fourier analysis (see Appendix A.1). As a first step, it allows us to formulate new and alternative representations for I- and IA estimators. The following theorem provides the representation of these estimators in the frequency domain.

**Theorem 1** 
(Fourier I- and IA-representation)**.** *Let $\overset{ˇ}{a}$ be an I- or IA estimator of a (cf. (4)) and let its (aperture) pull-in region $\Omega_{0}$ be symmetric about the origin. Then its Fourier representation is given as*

(5) $$\overset{ˇ}{a}=\hat{a}-\sum_{z\in Z^{n}}c\left(z\right)\sin\left(2\pi z^{T}\hat{a}\right)$$

*with Fourier coefficients $c\left(z\right)=\int_{\Omega_{0}}x\sin\left(2\pi z^{T}x\right)dx$.*

**Proof.**  See Appendix A.2. □

Noteworthy about this representation is that it provides a clear separation between the impact of the choice of aperture pull-in region $\Omega_{0}$ and the impact of the BLUE $\hat{a}$. The impact of $\Omega_{0}$ is completely confined to the Fourier coefficients, whereas the BLUE $\hat{a}$ only appears in the sine-arguments. In the one-dimensional case, for instance, one gets for the choice $\Omega_{0}=\left[-\frac{1}{2}\lambda ,+\frac{1}{2}\lambda \right]$, 0<λ≤1, the coefficients as $c\left(z\right)=\frac{1}{2}\lambda^{2}j_{1}\left(\pi \lambda z\right)$, with $j_{1}\left(x\right)=\frac{1}{x^{2}}\left(\sin\left(x\right)-x\cos\left(x\right)\right)$ being the spherical Bessel function of the first kind and order. It is an oscillating function that dies out quickly for increasing |z|, thus implying that only a finite terms in the sum are needed. Hence, due to the mentioned separation, expression (5) can be used for designing one’s own I or IA estimator through a direct specification of the Fourier coefficients. Also, for any such set of Fourier coefficients, one can quite easily analyze variations or pertubations in $\hat{a}$, as they simply appear as phase shifts. This can be useful, for instance, in the case of Monte Carlo simulations or when analyzing the impact of biases.

In the next sections we will develop the Fourier representations of GNSS ambiguity validation further for the method of IA-bootstrapping. This method, which was introduced in [13], has the advantage over other ambiguity validation methods [4,7,8,24,25] in that a complete analytical solution is available for its performance diagnostics. We will take advantage of this by providing these performance measures in spatial, frequency and hybrid domain.

## 3. Integer Aperture Bootstrapped (IAB) Estimation

### 3.1. The IAB Estimator

The IAB estimator is a generalization of the integer-bootstrapped (IB) estimator. The entries of the IB estimator ${\overset{ˇ}{a}}_{B}={\left({\overset{ˇ}{a}}_{B,1},\ldots ,{\overset{ˇ}{a}}_{B,n}\right)}^{T}\in Z^{n}$ are computed sequentially as follows:

(6) $$\left\{\begin{array}{ccccc} {\overset{ˇ}{a}}_{B,1} & = & \left\lfloor {\hat{a}}_{1}\right\rceil & & \\ {\overset{ˇ}{a}}_{B,2} & = & \left\lfloor {\hat{a}}_{2|1}\right\rceil & = & \left\lfloor {\hat{a}}_{2}-\sigma_{21}\sigma_{1}^{-2}\left({\hat{a}}_{1}-{\overset{ˇ}{a}}_{B,1}\right)\right\rceil \\ & \vdots & & & \\ {\overset{ˇ}{a}}_{B,n} & = & \left\lfloor {\hat{a}}_{n|N}\right\rceil & = & \left\lfloor {\hat{a}}_{n}-\sum_{j=1}^{n-1}\sigma_{n,j|J}\sigma_{j|J}^{-2}\left({\hat{a}}_{j|J}-{\overset{ˇ}{a}}_{B,j}\right)\right\rceil \end{array}\right.$$

where ‘⌊.⌉’ denotes rounding to the nearest integer, $\sigma_{i,j|J}$ denotes the covariance between ${\hat{a}}_{i}$ and ${\hat{a}}_{j|J}$, and $\sigma_{j|J}^{2}$ is the variance of ${\hat{a}}_{j|J}$. The shorthand notation ${\hat{a}}_{i|I}$ stands for the *i*th least-squares ambiguity obtained through a conditioning on the previous I={1,…,(i−1)} sequentially rounded ambiguities.

The IB estimator is thus a combination of sequential conditional least-squares estimation and integer rounding. When computing the IB-solution it is very useful to make use of the triangular factorization of the ambiguity variance matrix. Due to the close relationship that exists between sequential conditional least-squares estimation and the unique lower triangular factorization of the ambiguity variance matrix, $Q_{\hat{a}}=LDL^{T}$, we have the following statistical interpretation of the entries of *L* and *D*:

(7) $${\left(L\right)}_{ij}=\left\{\begin{array}{ccc} 0 & \mathrm{if} & 1\leq i<j\leq \;n\\ 1 & \mathrm{if} & i=j\\ \sigma_{i,j|J}\sigma_{j|J}^{-2} & \mathrm{if} & 1\leq j<i\leq n \end{array}\right.\;\mathrm{and}\;D=\mathrm{diag}\left(\ldots ,\sigma_{j|J}^{2},\ldots \right)$$

**Figure 2:**
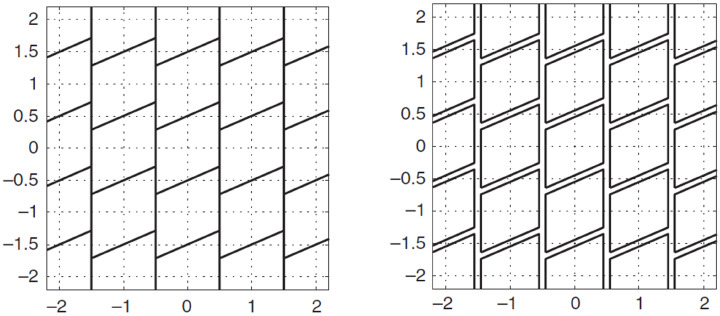
Two-dimensional pull-in regions $P_{\mathrm{IB},z}$ of integer bootstrapping (**left**) and two-dimensional aperture pull-in regions $\Omega_{z}=\lambda P_{\mathrm{IB},z}\subset P_{\mathrm{IB},z}$ of integer-aperture bootstrapping (**right**).

This shows that the coefficients needed in (6) are given by the lower triangular entries of *L*. The unit lower triangular matrix *L* can also be used to describe the IB pull-in regions. For the origin, the IB pull-in region is given as

(8) $$P_{\mathrm{IB},0}=\{x\in R^{n}\left|\;\right|c_{i}^{T}L^{-1}x\left|\leq \frac{1}{2},i=1,\ldots ,n\right\}$$

in which the *n*-vectors $c_{i}$ are canonical unit vectors with 1s as the *i*th entry and zeros otherwise. The IB pull-in regions are multivariate versions of parallellograms. An example is shown in Figure 2 (left) for the two-dimensional case.

The aperture pull-in region of IA-bootstrapping is a scaled version of the IB pull-in region,

(9) $$\Omega_{B,0}=\lambda P_{\mathrm{IB},0}=\left\{x\in R^{n}|\;\;x/\lambda \in P_{\mathrm{IB},0}\right\}$$

with 0<λ≤1. The shape of the pull-in region $\Omega_{B,z}$ is thus identical to that of the bootstrapped pull-in region $P_{\mathrm{IB},z}$. Only their sizes differ. By varying the aperture parameter λ one varies the size of $\Omega_{B,z}\subset P_{\mathrm{IB},z}$; see Figure 2 (right). Since both $\Omega_{B,z}$ and $P_{\mathrm{IB},z}$ have the same shape, the computation of the IA-bootstrapped estimator is almost as simple as that of the IB estimator. The computational steps are as follows. As before, one starts with the float solution $\hat{a}$ and computes the bootstrapped solution ${\overset{ˇ}{a}}_{B}$. This result identifies the aperture pull-in region $\Omega_{B,{\overset{ˇ}{a}}_{B}}$ for which it needs to be verified whether or not the float solution resides in it. Note that this verification is equivalent to the verification whether or not $\frac{1}{\lambda }\left(\hat{a}-{\overset{ˇ}{a}}_{B}\right)\in P_{\mathrm{IB},0}$. Thus, from $\hat{a}$ and ${\overset{ˇ}{a}}_{B}$, one forms the bootstrapped ambiguity residual ${\overset{ˇ}{\epsilon }}_{B}=\hat{a}-{\overset{ˇ}{a}}_{B}$, up-scales it to $\frac{1}{\lambda }{\overset{ˇ}{\epsilon }}_{B}$ and verifies whether this up-scaled version still resides in $P_{\mathrm{IB},0}$. This is carried out by using the same bootstrapping procedure as before, but now applied to the input $\frac{1}{\lambda }{\overset{ˇ}{\epsilon }}_{B}$. If the outcome is the zero vector, then the IAB-outcome is ${\overset{ˇ}{a}}_{B}$; otherwise it is $\hat{a}$.

The conclusion therefore shows that the computation of the IAB estimator $\overset{ˇ}{a}$ is thus very simple indeed. It essentially consists of applying the bootstrapped procedure twice, once to the float solution $\hat{a}$ and once to the upscaled residual $\frac{1}{\lambda }{\overset{ˇ}{\epsilon }}_{B}$. 

### 3.2. Performance of the IAB Estimator in Spatial Domain

In order to evaluate the performance of an IA estimator, it is helpful to first classify its possible outcomes. An IA estimator can produce one of the following three outcomes: a correct outcome when $\overset{ˇ}{a}=a\in Z^{n}$, an incorrect outcome when $\overset{ˇ}{a}=z\in Z^{n}\setminus \left\{a\right\}$, and a no-integer outcome when $\overset{ˇ}{a}=\hat{a}\in R^{n}\setminus \left\{Z^{n}\right\}$. A correct integer outcome may be considered a *success*, an incorrect integer outcome a *failure*, and an outcome where no correction at all is given to the float solution $\hat{a}$ as indeterminate or *undecided*. The probability of success, the *success rate* $P_{S}$, equals the integral of the PDF of the float solution, $f_{\hat{a}}\left(x\right)$, over $\Omega_{a}$, whereas the probability of failure, the *fail rate* $P_{F}$, equals the integral of $f_{\hat{a}}\left(x\right)$ over $\Omega \setminus \Omega_{a}$. The respective probabilities are therefore given as

(10) $$\left\{\begin{array}{ccccc} P_{S} & = & P(\overset{ˇ}{a}=a) & = & \int_{\Omega_{a}}f_{\hat{a}}\left(x\right)dx\;\;\;\left(\mathrm{success}\right)\\ P_{F} & = & \sum_{z\neq a}P\left(\overset{ˇ}{a}=z\right) & = & \sum_{z\neq a}\int_{\Omega_{z}}f_{\hat{a}}\left(x\right)dx\;\;\;\left(\mathrm{failure}\right)\\ P_{U} & = & P(\overset{ˇ}{a}=\hat{a}) & = & 1-P_{S}-P_{F}\;\;\;\left(\mathrm{undecided}\right) \end{array}\right.$$

**Figure 3:**
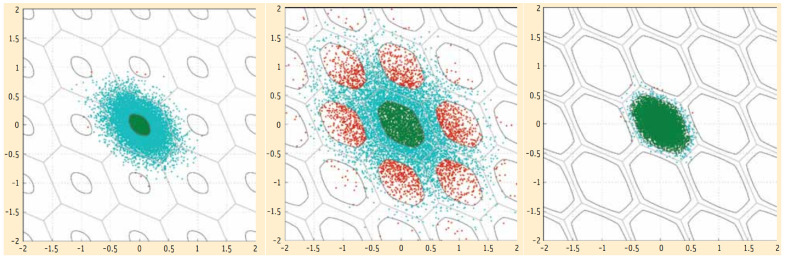
Blue scatter plots of samples of $\hat{a}$ at three different precision levels (poor precision at **left** and in **middle**, good precision at **right**), showing those that lead to correct IA-outcomes as green ($\hat{a}\in \Omega_{a}$) and those to incorrect IA-outcomes as red ($\hat{a}\in \Omega_{z\neq a}$). Aperture pull-in regions $\Omega_{z}$ at the **left** and **right** are properly scaled, while those in the middle are not.

Note that these three probabilities are completely governed by $f_{\hat{a}}\left(x\right)$, the PDF of the float solution $\hat{a}$, and by $\Omega_{0}$, the aperture pull-in region, which uniquely defines the IA estimator. By setting the size and shape of the aperture pull-in region $\Omega_{0}\subset P_{0}$ for a given PDF $f_{\hat{a}}\left(x\right)$, the user has now gained control on the level of the fail rate they are willing to accept. By controlling the fail rate $P_{F}$ through a proper choice of $\Omega_{0}$, the user can make sure to have a large enough successful fixing rate

(11) $$P_{\mathrm{SF}}=\frac{P_{S}}{P_{S}+P_{F}}$$

and thus provide enough confidence in the integer outcomes of IA-estimation. This is illustrated for three different cases in Figure 3. The blue scatter plots are those of the BLUE $\hat{a}$, with, on the left and right, a proper scaling of the aperture pull-in regions, while in the middle, the pull-in regions have a too large aperture, a consequence of which is that the fail rate is too large as well.

We will now determine these probabilities for the IAB estimator. Apart from the ease with which this estimator can be computed, it also has the user-advantage that analytical closed form expressions can be given for its fail rate $P_{F}$ and its success rate $P_{S}$. We have the following theorem due to [13].

**Theorem 2** 
(The IAB performance probabilities)**.** *Let the float solution be distributed as $\hat{a}∼N_{n}\left(a,Q_{\hat{a}\hat{a}}\right)$, $a\in Z^{n}$, and let its variance matrix have the unique triangular factorization $Q_{\hat{a}\hat{a}}=LDL^{T}$, with L a unit lower triangular matrix and D a diagonal matrix. The IAB-probabilities of integer estimation, $P_{I}\left(\lambda \right)=P_{S}\left(\lambda \right)+P_{F}\left(\lambda \right)$, and successful integer estimation, $P_{S}\left(\lambda \right)$, are then given as*

(12) $$P_{S}\left(\lambda \right)=\prod_{i=1}^{n}p_{\lambda ,\sigma_{i|I}}\left(0\right)\;\;\mathrm{and}\;\;P_{I}\left(\lambda \right)=\sum_{z\in Z^{n}}\prod_{i=1}^{n}p_{\lambda ,\sigma_{i|I}}\left(c_{i}^{T}L^{-1}z\right)$$

*with*

(13) $$p_{\lambda ,\sigma }\left(s\right)=\Phi \left(\frac{\lambda -2s}{2\sigma }\right)+\Phi \left(\frac{\lambda +2s}{2\sigma }\right)-1$$

*and where λ is the aperture parameter (0<λ≤1), $\sigma_{i|I}^{2}={\left(D\right)}_{ii}$, $\Phi \left(x\right)=\int_{-\infty }^{x}\frac{1}{\sqrt{2\pi }}\exp\left\{-\frac{1}{2}v^{2}\right\}dv$ and $c_{i}$ denotes the canonical unit vector having as its ith entry a 1 and zeros otherwise.*

Recall that the aperture pull-in region is defined as a λ-scaled version of the bootstrapped pull-in region, $\Omega_{B,0}=\lambda P_{\mathrm{IB},0}$. The above result will therefore reduce to that of the IB estimator when the aperture parameter is set equal to 1. In that case, $P_{S}$ becomes identical to the success rate of integer bootstrapping and $P_{F}=1-P_{S}$, since $P_{U}=0$. This maximum value of λ is acceptable if the corresponding fail rate $P_{F}$ is at a small enough level for the successful fixing rate $P_{\mathrm{SF}}$ to be large enough. As this is seldom the case in practice, the need for ambiguity validation by means of the analytically accessible IAB estimator remains a necessity.

The above result shows, as is the case with integer bootstrapping, that the IAB-probabilities are driven by the entries of the diagonal matrix *D*, i.e., by the spectrum of sequential conditional variances $\sigma_{i|I}^{2}$, i=1,…,n. Therefore GNSS ambiguity parametrizations are used in practice and aim at a sufficiently flat spectrum such that $\sigma_{i|I}≲\sigma_{i+1|I+1}$, thus showing that integer bootstrapping is done on the most precise ambiguities first [21,22].

Although the proof of (12) can already be found in [13], an alternative proof will be given here such that it can also be used as starting point for the development of its frequency representation. As the probability $P_{I}\left(\lambda \right)=P_{S}\left(\lambda \right)+P_{F}\left(\lambda \right)$ is the sum of all the probabilities that $\hat{a}$ lies in one of the aperture pull-in regions $\Omega_{z}=\lambda P_{\mathrm{IB},z}$, we have

(14) $$\begin{array}{ccc} P_{I}\left(\lambda \right) & = & \sum_{z\in Z^{n}}P\left[\hat{a}\in \lambda P_{\mathrm{IB},z}\right]\\ & \overset{\left(1\right)}{=} & \sum_{z\in Z^{n}}P\left[\hat{a}-z\in \lambda P_{\mathrm{IB},0}\right]\\ & \overset{\left(2\right)}{=} & \sum_{z\in Z^{n}}P\left[L^{-1}\left(\hat{a}-a+z\right)\in \lambda L^{-1}P_{\mathrm{IB},0}\right]\\ & \overset{\left(3\right)}{=} & \sum_{z\in Z^{n}}P\left[u+L^{-1}z\in \lambda C_{0}^{n}\right] \end{array}$$

with $u∼N_{n}\left(0,D\right)$ and $C_{0}^{n}={\left[-\frac{1}{2},+\frac{1}{2}\right]}^{n}$ being the origin-centred unit-cube. Step (1) follows, since $\lambda P_{\mathrm{IB},z}=\lambda P_{\mathrm{IB},0}+z$. In step (2), the subtraction of the integer constant *a* and the change in sign of *z*, leaves the sum over all integers of $Z^{n}$ unchanged. Furthermore, its multiplication with $L^{-1}$, results in $u=L^{-1}\left(\hat{a}-a\right)$ being zero-mean Gaussian distributed with diagonal variance matrix *D*. In step (3), we recognize that the transformation by $L^{-1}$ of the IB pull-in region $P_{\mathrm{IB},0}=\{x\in R^{n}\left|\;\right|c_{i}^{T}L^{-1}x\left|\leq \frac{1}{2},i=1,\ldots ,n\right\}$, results in the unit-cube $C_{0}^{n}=\{x\in R^{n}\left|\;\right|c_{i}^{T}x\left|\leq \frac{1}{2},i=1,\ldots ,n\right\}$. As the components of *u* are independent, the last expression of (14) can be written as a sum of products, $P_{I}\left(\lambda \right)=\sum_{z\in Z^{n}}\prod_{i=1}^{n}P\left[-\frac{1}{2}\lambda \leq u_{i}+c_{i}^{T}L^{-1}z\leq \frac{1}{2}\lambda \right]$, from which then the result of Theorem 2 follows, thereby noting that the z=0 term of the sum gives $P_{S}$ and the sum of the remaining z≠0 terms gives $P_{F}$.

### 3.3. Performance of the IAB Estimator in Frequency Domain

We will now develop the frequency representation of the performance probabilities of the IAB estimator. We will do this for the sum $P_{I}\left(\lambda \right)=P_{S}\left(\lambda \right)+P_{F}\left(\lambda \right)$, as the corresponding expression for the fail rate simply follows from subtracting the success rate (cf. (12)) from it. For that purpose, we first state and prove the following lemma.

**Lemma 1.** 
*Let f(x) and F(s) each be the other’s Fourier pair. Then, for any $b\in R^{n}$ and any invertible matrix $B\in R^{n\times n}$,*

(15) $$\sum_{z\in Z^{n}}f\left(x+b+Bz\right)={\left|B\right|}^{-1}\sum_{z\in Z^{n}}c\left(z\right)\exp\left(2\pi j{\left(B^{-T}z\right)}^{T}x\right)$$

*with coefficients $c\left(z\right)=F\left(B^{-T}z\right)\exp\left(2\pi j{\left(B^{-T}z\right)}^{T}b\right)$.*

**Proof.**  See Appendix A.3. □

This result can be considered a generalization of the well-known Poisson summation formula [26,27]. Poisson’s summation formula follows from (15) as $\sum_{z\in Z^{n}}f\left(x+z\right)=\sum_{z\in Z^{n}}F\left(z\right)\exp\left(2\pi jz^{T}x\right)$, when b=0 and $B=I_{n}$.

With (15), we are now in a position to develop the frequency representation of $P_{I}\left(\lambda \right)$. Starting from the last expression of (14), denoting the PDF of $u∼N_{n}\left(0,D\right)$ as $f_{u}\left(x\right)$ and its Fourier transform as $F_{u}\left(s\right)$, we may write

(16) $$\begin{array}{ccc} P_{I}\left(\lambda \right) & = & \sum_{z\in Z^{n}}P\left[u+L^{-1}z\in \lambda C_{0}^{n}\right]\\ & \overset{\left(1\right)}{=} & \int_{\lambda C_{0}^{n}}\sum_{z\in Z^{n}}f_{u}\left(x+L^{-1}z\right)dx\\ & \overset{\left(2\right)}{=} & \left|L\right|\sum_{z\in Z^{n}}F_{u}\left(L^{T}z\right)\int_{\lambda C_{0}^{n}}\exp\left(2\pi j{\left(L^{T}z\right)}^{T}x\right)dx\\ & \overset{\left(3\right)}{=} & \sum_{z\in Z^{n}}F_{u}\left(L^{T}z\right)\int_{\lambda C_{0}^{n}}\prod_{i=1}^{n}\exp\left(2\pi j\left(c_{i}^{T}L^{T}z\right)x_{i}\right)dx_{i}\\ & \overset{\left(4\right)}{=} & \sum_{z\in Z^{n}}F_{u}\left(L^{T}z\right)\prod_{i=1}^{n}\int_{-\lambda /2}^{+\lambda /2}\exp\left(2\pi j\left(c_{i}^{T}L^{T}z\right)x_{i}\right)dx_{i}\\ & \overset{\left(5\right)}{=} & \sum_{z\in Z^{n}}F_{u}\left(L^{T}z\right)\prod_{i=1}^{n}\int_{-\lambda /2}^{+\lambda /2}\cos\left(2\pi \left(c_{i}^{T}L^{T}z\right)x_{i}\right)dx_{i}\\ & \overset{\left(6\right)}{=} & \sum_{z\in Z^{n}}F_{u}\left(L^{T}z\right)\prod_{i=1}^{n}\lambda \frac{\sin\;(\pi \lambda c_{i}^{T}L^{T}z)}{\pi \lambda c_{i}^{T}L^{T}z} \end{array}$$

In step (1), we used the PDF of *u*, $f_{u}\left(x\right)$, to express the probability as an integral over the scaled unit cube. This is followed by an application of Lemma 1 in step (2). In step (3) we used the fact that the determinant of the unit triangular matrix *L* equals 1 and that the exponential of a sum of terms can be written as a product of exponentials in these terms. In step (4) we expressed the multivariate integral over the scaled unit-cube as a product of scalar integrals over the interval $\left[-\frac{1}{2}\lambda ,+\frac{1}{2}\lambda \right]$. As this integration interval is symmetric with respect to the origin and the imaginary part of the exponential an odd function, we are left in step (5) only with the real-valued cosine functions. Integration of these cosine functions produces the sinc-functions in step (6).

Note that the steps of (16) are generally valid, as they do not rely on any specific assumptions about the distribution of *u* other than stating that $f_{u}\left(x\right)$ and $F_{u}\left(s\right)$ form a Fourier-pair. Hence, this result is also useful when working with larger classes of distributions, like, for instance, the elliptically contoured class [28,29]. In our present case, however, we are working with normally distributed variables and have $f_{u}\left(x\right)={\left(2\pi \right)}^{-n/2}{\left|D\right|}^{-1/2}\exp\left\{-\frac{1}{2}x^{T}D^{-1}x\right\}$, since $u∼N_{n}\left(0,D\right)$. The Fourier transform of this $f_{u}\left(x\right)$ is known as the characteristic function [30,31] and given as $F_{u}\left(s\right)=\exp\left(-\frac{1}{2}4\pi^{2}z^{T}Dz\right)$. Substitution of this characteristic function into (16) concludes the proof of the following theorem.

**Theorem 3** 
(IAB performance probability in frequency domain)**.** *Under the same assumptions of Theorem 2, the IAB probability of integer estimation $P_{I}\left(\lambda \right)=P_{S}\left(\lambda \right)+P_{F}\left(\lambda \right)$ has the frequency representation*

(17) $$P_{I}\left(\lambda \right)=\sum_{z\in Z^{n}}c\left(z\right)\prod_{i=1}^{n}q_{\lambda }\left(c_{i}^{T}L^{T}z\right)$$

*with*

(18) $$c\left(z\right)=\exp(-\frac{1}{2}4\pi^{2}\left|\right|L^{T}{z||}_{D^{-1}}^{2})\;\;\mathrm{and}\;\;q_{\lambda }\left(s\right)=\lambda \frac{\sin\;(\pi \lambda s)}{\pi \lambda s}$$

With this result, we now have two different expressions available for the IAB-probability $P_{I}\left(\lambda \right)$, in the spatial domain (12) and in the frequency domain (17). To assess their relative merits, observe that both involve infinite sums over the entire set of integers, which is clearly computationally impractical. Nevertheless, sufficient accuracy can be achieved by replacing these infinite sums with finite sums over integers selected from sufficiently large search ellipsoids [19]. It is at this point that the distinction between the two expressions becomes apparent. In the spatial representation, integers are collected according to inequality $\left|\right|\hat{a}-{z||}_{Q_{\hat{a}\hat{a}}}^{2}=\left|\right|L^{-1}\left(\hat{a}-z\right){\left|\right|}_{D}^{2}\leq r^{2}$, whereas in the frequency representation, inequality ${\left||z|\right|}_{Q_{\hat{a}\hat{a}}^{-1}}^{2}=\left|\right|L^{T}z{\left|\right|}_{D^{-1}}^{2}\leq \rho^{2}$ is applied. This indicates that the spatial representation is advantageous when the ambiguities are precise, i.e., when $Q_{\hat{a}\hat{a}}$ (or *D*) is small, while the frequency representation is preferable when the ambiguities are poorly determined, i.e., when $Q_{\hat{a}\hat{a}}^{-1}$ (or $D^{-1}$) is small.

Even if the ambiguity precision itself does not determine the choice between the two representations, one may sometimes still prefer the frequency representation. This is particularly relevant in Monte Carlo studies where a large number of $\hat{a}$-samples are generated and a choice must be made between the two representations. Since the frequency coefficients c(z) (cf. (18)) then need to be computed only once, and because repeated evaluations of the sine and cosine of a dot product are generally less expensive than repeatedly computing the exponential of a quadratic form with a dense variance matrix, the use of (17) will then be favored. At this point we also remark that one can take advantage of the symmetry in (17): Note, since $q_{\lambda }\left(s\right)$ is an even function and $\mathrm{sinc}\left(\lambda s\right)=q_{\lambda }\left(s\right)/\lambda$ has the limit $\lim_{x\to 0}\mathrm{sinc}\left(x\right)=1$, that (17) can be written as

(19) $$P_{I}\left(\lambda \right)=\lambda^{n}+2\sum_{z\in Z_{+}^{n}}c\left(z\right)\prod_{i=1}^{n}q_{\lambda }\left(c_{i}^{T}L^{T}z\right)$$

in which $Z_{+}^{n}$ is the half grid, taking only one of each nonzero integer pair {−z,+z}.

Although the preceding theorem clearly indicates which representation should be preferred depending on the ambiguity precision, its direct practical applicability remains limited. In practice, it is uncommon for all ambiguities to be either uniformly precise or uniformly imprecise. Consequently, a more flexible approach is required—one that can accommodate ambiguity sets containing both precise and less precise components. This flexibility is provided by our hybrid formulation introduced in the next section.

## 4. IAB Performance Probability in Hybrid Form

The need for a hybrid formulation stems from the computational complexity that a IAB-probability formulation in the spatial domain poses when the dimension *n* increases. The integers used in the summation over $Z^{n}$ are usually obtained from a search ellipsoid $E_{\hat{a}}=\{z\in Z^{n}\left||\right|\hat{a}-{z||}_{Q_{\hat{a}\hat{a}}}^{2}\leq r^{2}=\chi_{\alpha }^{2}\left(n,0\right)\}$ in which α is a user-defined significance level of the central Chi-square distribution with *n* degrees of freedom [22]. As the volume of the ellipsoid is a good rule of thumb for the number of integer candidates contained in it, one can infer, for less precise ambiguities, that the required number of integer vectors increases dramatically with its dimension. Using asymptotics for the volume of $E_{\hat{a}}$, the number of integer candidates can be approximated as $f\left(n\right){\left(\mathrm{ADOP}\right)}^{n}$ [32], in which $f\left(n\right)=\frac{{\left(2\pi e\right)}^{n/2}}{\sqrt{\pi n}}$ shows, unless the ADOP is small enough, how the number of integer candidates explodes as a function of the dimension *n*. We have, for instance, $f\left(5\right)=3\times 10^{2}$, $f\left(10\right)=2.6\times 10^{5}$, and $f\left(15\right)=2.6\times 10^{8}$. This identified “dimensional curse” raises concerns about the applicability of the spatial IAB-probability formulation in modern-day GNSS. Whereas earlier applications often relied solely on GPS, current practice predominantly employs multi-frequency, multi-GNSS approaches [1,3,5,33,34,35,36,37], thereby combining the observation equations of GPS, GLONASS, Galileo, and BeiDou. This effectively quadruples the number of ambiguities per observed frequency, thus putting a significantly increased computational load on the crucial step of carrier-phase GNSS ambiguity validation. With the future integration of mega-constellations of LEO satellites, the situation is expected to become even more demanding [38,39].

However, switching from the spatial representation to the all-frequency representation is not a universally viable solution. In many cases, this alternative becomes numerically problematic due to the high precision typically associated with a substantial subset of the ambiguities. Consequently, overcoming the “dimensional curse” requires a hybrid formulation of the IAB performance probabilities, the solution of which is presented in the following theorem.

**Theorem 4** 
(IAB performance probability in hybrid form)**.** *Let $\hat{a}∼N_{n}\left(a,Q_{\hat{a}\hat{a}}=LDL^{T}\right)$ have the partitioning*

(20) $$a=\left[\begin{array}{c} a_{1}\in Z^{n_{1}}\\ a_{2}\in Z^{n_{2}} \end{array}\right],L=\left[\begin{array}{cc} L_{11} & 0\\ L_{21} & L_{22} \end{array}\right],D=\left[\begin{array}{cc} D_{1} & 0\\ 0 & D_{2} \end{array}\right]$$

*with $D_{1}=\mathrm{diag}\left(\sigma_{1}^{2},\sigma_{2|1}^{2},\ldots ,\sigma_{n_{1}|N_{1}}^{2}\right)$, $D_{2}=\mathrm{diag}\left(\sigma_{n_{1}+1|N_{1}+1}^{2},\ldots ,\sigma_{n|N}^{2}\right)$. Then the hybrid spatial-frequency representation of the IAB probability of integer estimation $P_{I}\left(\lambda \right)=P_{S}\left(\lambda \right)+P_{F}\left(\lambda \right)$ is given as*

(21) $$P_{I}\left(\lambda \right)=\sum_{z_{1}\in Z^{n_{1}}}F_{\lambda }\left(z_{1}\right)\sum_{z_{2}\in Z^{n_{2}}}G_{\lambda }\left(z_{2}\right)\cos\left(2\pi z_{2}^{T}L_{21}L_{11}^{-1}z_{1}\right)$$

*with*

(22) $$\begin{array}{ccc} F_{\lambda }\left(z_{1}\right) & = & \prod_{i=1}^{n_{1}}p_{\lambda ,\sigma_{i|I}}\left(c_{i}^{T}L_{11}^{-1}z_{1}\right)\\ G_{\lambda }\left(z_{2}\right) & = & c\left(z_{2}\right)\prod_{i=n_{1}+1}^{n}q_{\lambda }\left(c_{i}^{T}L_{22}^{T}z_{2}\right)\\ c(z_{2}) & = & \exp(-\frac{1}{2}4\pi^{2}||L_{22}^{T}z_{2}|{|}_{D_{2}^{-1}}^{2}) \end{array}$$

**Proof.**  See Appendix A.4. □

The above results are expressed in the entries of *L* and *D*. Note, however, since $Q_{{\hat{a}}_{1}{\hat{a}}_{1}}=L_{11}D_{1}L_{11}^{T}$, $Q_{{\hat{a}}_{2}\left|a_{1}{\hat{a}}_{1}\right|a_{1}}=L_{22}D_{2}L_{22}^{T}$, and $Q_{{\hat{a}}_{2}{\hat{a}}_{1}}Q_{{\hat{a}}_{1}{\hat{a}}_{1}}^{-1}=L_{21}L_{11}^{-1}$, how the precisions of ${\hat{a}}_{1}$ and ${\hat{a}}_{2}|a_{1}$ drive the functions $F_{\lambda }\left(z_{1}\right)$ and $G_{\lambda }\left(z_{2}\right)$, respectively, while their covariance takes care of the coupling between the two sums over $Z^{n_{1}}$ and $Z^{n_{2}}$, respectively. When $Q_{21}=0$, the $z_{1}$-$z_{2}$ coupling disappears and we have the spatial-frequency product $P_{I}\left(\lambda \right)=\left(\sum_{z_{1}\in Z^{n_{1}}}F_{\lambda }\left(z_{1}\right)\right)\left(\sum_{z_{2}\in Z^{n_{2}}}G_{\lambda }\left(z_{2}\right)\right)$. Also note that, as an analogy of (19), the probability (21) can be written as $P_{I}\left(\lambda \right)=\sum_{z_{1}\in Z^{n_{1}}}F_{\lambda }\left(z_{1}\right)\left(\lambda^{n}+2\sum_{z_{2}\in Z_{+}^{n_{2}}}G_{\lambda }\left(z_{2}\right)\cos\left(2\pi z_{2}^{T}L_{21}L_{11}^{-1}z_{1}\right)\right)$. This now clearly shows the computational advantage the hybrid formulation brings. As $F_{\lambda }\left(z\right)$ is in the spatial domain and $G_{\lambda }\left(z\right)$ in the frequency domain, both die out quickly if $Q_{{\hat{a}}_{1}{\hat{a}}_{1}}$ is small and $Q_{{\hat{a}}_{2}\left|a_{1}{\hat{a}}_{1}\right|a_{1}}$ large. This would not happen however, if the full spatial domain representation is used. The poor precision of ${\hat{a}}_{2}$ would then hinder fast convergence.

The steps for computing the IAB performance probability are thus as follows. From computing the triangular factorization of the ambiguity variance matrix, $Q_{\hat{a}\hat{a}}=LDL^{T}$ (preferably after LAMBDA decorrelation, using e.g., the toolbox of [22]), one obtains, from the diagonal entries of matrix *D*, the spectrum of sequential conditional variances $\sigma_{i|I}^{2}$, i=1,…,n. If the spectrum is flat, one uses the full spatial representation (cf. (12)) when the ADOP is small (e.g., <0.2 cycles), but the full spectral representation (cf. (17)) when the ADOP is not. For cases where the spectrum shows a discontinuity, the hybrid representation is used by allocating the spatial and frequency parts to the appropriate parts of the spectrum.

To illustrate the workings of the hybrid theorem and to show the computational advantage it brings when the spectrum of sequential conditional ambiguity variances vary, we consider a three-dimensional example with $\hat{a}={\left(0,0,0\right)}^{T}$ as a float solution and for its variance matrix $Q_{\hat{a}\hat{a}}=LDL^{T}$, the following triangular factors,

(23) $$L=\left[\begin{array}{ccc} 1 & 0 & 0\\ 0.7 & 1 & 0\\ -0.3 & 0.4 & 1 \end{array}\right],\;D=\left[\begin{array}{ccc} 0.01 & 0 & 0\\ 0 & 0.2 & 0\\ 0 & 0 & 10 \end{array}\right]$$

We compare the following three approaches for truncation: full-spatial, full-frequency, and hybrid spatial-frequency truncation for λ=0.6.

*Full-spatial:* To reach an absolute accuracy of about $10^{-12}$ in the sum, the conducted ellipsoidal search of $z^{T}Q_{\hat{a}\hat{a}}^{-1}z={\left(L^{-1}z\right)}^{T}D^{-1}\left(L^{-1}z\right)\leq r^{2}$ returns, for r=8, a total of 285 integer vectors.
*Full-frequency:* For a similar accuracy, the ellipsoidal search of $z^{T}Q_{\hat{a}\hat{a}}z={\left(L^{T}z\right)}^{T}D\left(L^{T}z\right)\leq \rho^{2}$, returns, for ρ=1.2, a total of 93 integer vectors.
*Hybrid spatial-frequency:* As the third conditional variance is much larger than the first two, the hybrid formulation is 2D spatial and 1D spectral. The two-dimensional spatial ellipsoidal search space is given as ${\left(z_{1}/\sigma_{1}\right)}^{2}+{\left(\left[z_{2}-0.7z_{2}\right]/\sigma_{2|1}\right)}^{2}\leq r^{2}$, which, for the same numerical accuracy, now only contains the integer set $\left(z_{1},z_{2}\right)\in \left\{\left(0,0\right),\left(0,\pm 1\right),\left(0,\pm 2\right),\left(0,\pm 3\right)\right\}$. This is due to the exclusion of the relatively large value $\sigma_{3|1,2}^{2}=10$, which also makes the frequency sum very efficient, namely requiring only one term, $z_{3}=0$. So in this example, the hybrid formulation only needs a sum over seven three-dimensional integer vectors, whereas the full-spatial and full-frequency formulations would need sums over 285 and 93 three-dimensional integer vectors, respectively.

## 5. Summary and Conclusions

Carrier-phase ambiguity validation is essential for ensuring the reliability of high-precision GNSS positioning. By formulating validation within the class of integer aperture (IA) estimators, the decision to fix or withhold integer ambiguities becomes an intrinsic part of the estimation framework. IA estimators, situated between integer and integer-equivariant (IE) estimators, introduce adjustable acceptance regions in the float ambiguity domain and thereby enable a controlled trade-off between fixing efficiency and reliability.

In this contribution, a general Fourier representation of IA estimators was established by exploiting the $Z^{n}$-periodic structure of ambiguity residuals. This frequency-domain formulation complements the conventional spatial description and provides additional analytical insight into the behavior of ambiguity validation procedures. Particular emphasis was placed on integer-aperture bootstrapping (IAB), for which explicit expressions of the success and failure probabilities were derived and shown to depend on the spectrum of sequential conditional ambiguity variances. A generalized Poisson summation approach enabled these probabilistic characteristics to be expressed in the frequency domain as well.

Since modern multi-GNSS applications involve ambiguity sets with heterogeneous precision levels, neither a purely spatial nor a purely spectral representation is sufficient on its own. The spatial, frequency, and hybrid formulations were therefore unified into a flexible framework capable of accommodating realistic ambiguity configurations. Taken together, the results provide a coherent spatial–spectral perspective on carrier-phase ambiguity validation and strengthen its theoretical and practical foundation within integer aperture estimation.

## Data Availability

All data generated or analyzed during this study are included in this contribution.

## Appendix A

### Appendix A.1. Multivariate Fourier Theory

Although Fourier theory is usually presented for scalar functions, the generalization to the multidimensional case, using vector functions, goes quite naturally. Some first results of Fourier theory are summarized here. Let each of the two multivariate functions $f,F:R^{n}\to C$ be the other’s Fourier transform pair. Then

(A1) $$\begin{array}{c} F\left(s\right)=\int_{R^{n}}f\left(x\right)\exp\left(-j2\pi s^{T}x\right)dx\\ f\left(x\right)=\int_{R^{n}}F\left(s\right)\exp\left(+j2\pi s^{T}x\right)ds \end{array}$$

with *j* denoting the imaginary unit ($j^{2}=-1$). Next to the above Fourier pair, we have for functions $g:R^{n}\to C$ that are $Z^{n}$-periodic, g(x+z)=g(x), the following Fourier pair,

(A2) $$\begin{array}{c} G_{z}=\int_{P_{0}}g\left(x\right)\exp\left(-j2\pi z^{T}x\right)dx\\ g\left(x\right)=\sum_{z\in Z^{n}}G_{z}\exp\left(+j2\pi z^{T}x\right) \end{array}$$

This result is valid for any pull-in region $P_{0}$. If needed, Euler’s formula can be used to decompose the complex exponential in terms of cosines and sines, $\exp\left(+j2\pi \alpha \right)=e^{+j2\pi \alpha }=\cos\left(2\pi \alpha \right)+j\sin\left(2\pi \alpha \right)$. For proofs of the above pairs, we refer to textbooks such as e.g., [26,27,40].

### Appendix A.2. Proof Theorem 1 (Fourier I- and IA- Representation)

The ambiguity residual follows from (4) as $\hat{a}-\overset{ˇ}{a}=\sum_{z\in Z^{n}}\left(\hat{a}-z\right)\omega_{z}\left(\hat{a}\right)$. As this function is $Z^{n}$-periodic, we determine its Fourier coefficients as

(A3) $$\begin{array}{ccc} A_{z} & = & \int_{P_{0}}\sum_{u\in Z^{n}}\left(x-u\right)\omega_{u}\left(x\right)\exp\left(-2\pi jz^{T}x\right)dx\\ & \overset{\left(1\right)}{=} & \sum_{u}\int_{P_{0}}v\omega_{0}\left(v\right)\exp\left(-2\pi jz^{T}\left(v+u\right)\right)dv\\ & \overset{\left(2\right)}{=} & \int_{R^{n}}v\omega_{0}\left(v\right)\exp\left(-2\pi jz^{T}v\right)dv\\ & \overset{\left(3\right)}{=} & \int_{\Omega_{0}}v\exp\left(-2\pi jz^{T}v\right)dv \end{array}$$

Step (1) follows from the change in variables v=x−u, while step (2) uses the fact that the pull-in regions partition $R^{n}$ completely and $\exp(-2\pi jz^{T}u)=1$. In step (3), the properties of the indicator function $\omega_{0}\left(v\right)$ are used. From the last expression of (A3), it follows, since $\Omega_{0}$ is origin-symmetric, that $A_{z}=0$ for z=0 and that for z≠0, $A_{-z}=-A_{z}$. The latter shows that in the above expression for $A_{z}$ the cosine part cancels and only the sine part remains, thus giving the imaginary $A_{z}=-j\int_{\Omega_{0}}v\sin\left(2\pi z^{T}v\right)dv$. Substitution into the Fourier series $\hat{a}-\overset{ˇ}{a}=\sum_{z\in Z^{n}}A_{z}\exp\left(+2\pi jz^{T}\hat{a}\right)$ gives the result. The result holds true under the standard understanding that the Fourier series represents the function almost-everywhere in the mean-square sense.

### Appendix A.3. Proof of Lemma 1

If f(x) and F(s) are each other’s Fourier pairs, then it is not difficult to show with the aid of (A1) that f(Bx) and ${\left|B\right|}^{-1}F\left(B^{-T}s\right)$ are also each other’s Fourier pairs. We now show that ${\left|B\right|}^{-1}F\left(B^{-T}z\right)$ are the Fourier coefficients of the $Z^{n}$-periodic function g(Bx), when $g\left(x\right)=\sum_{z\in Z^{n}}f\left(x+Bz\right)$. We have

(A4) $$\begin{array}{ccc} \int_{P_{0}}g\left(Bx\right)\exp\left(-2\pi jz^{T}x\right)dx & = & \sum_{u\in Z^{n}}\int_{P_{0}}f\left(B\left(x+u\right)\right)\exp\left(-2\pi jz^{T}x\right)dx\\ & \overset{\left(1\right)}{=} & \sum_{u\in Z^{n}}\int_{P_{u}}f\left(Bv\right)\exp\left(-2\pi jz^{T}\left(v-u\right)\right)dv\\ & \overset{\left(2\right)}{=} & \int_{R^{n}}f\left(Bv\right)\exp\left(-2\pi jz^{T}v\right)dv\\ & \overset{\left(3\right)}{=} & {\left|B\right|}^{-1}F\left(B^{-T}z\right) \end{array}$$

Step (1) follows from the change of variables v=x+u, while step (2) uses the fact that the pull-in regions partition $R^{n}$ completely and $\exp(-2\pi jz^{T}u)=1$. In step (3), we used the earlier determined fact that f(Bx) and ${\left|B\right|}^{-1}F\left(B^{-T}s\right)$ are each other’s Fourier pairs. As (A4) shows that ${\left|B\right|}^{-1}F\left(B^{-T}z\right)$ are the Fourier coefficients of g(Bx), we have $g\left(Bx\right)=\sum_{z\in Z^{n}}{\left|B\right|}^{-1}F\left(B^{-T}z\right)\exp\left(+2\pi jz^{T}x\right)$, from which the result follows if we replace Bx by x+b.

### Appendix A.4. Proof of Theorem 4 (IAB Performance Probability in Hybrid Form)

With partitioning (20) and the independence of $u_{1}$ and $u_{2}$ in $u={\left(u_{1}^{T},u_{2}^{T}\right)}^{T}$, we may write the first expression of (16) using the results of Theorem 2 and $b\left(z_{1}\right)=-L_{22}^{-1}L_{21}L_{11}^{-1}z_{1}$, as

(A5) $$\begin{array}{ccc} P_{I}\left(\lambda \right) & = & \sum_{z\in Z^{n}}P\left[u+L^{-1}z\in \lambda C_{0}^{n}\right]\\ & = & \sum_{z_{1}\in Z^{n_{1}}}P\left[u_{1}+L_{11}^{-1}z_{1}\in \lambda C_{0}^{n_{1}}\right]\left(\sum_{z_{2}\in Z^{n_{2}}}P\left[u_{2}+b\left(z_{1}\right)+L_{22}^{-1}z_{2}\in \lambda C_{0}^{n_{2}}\right]\right)\\ & = & \sum_{z_{1}\in Z^{n_{1}}}F_{\lambda }\left(z_{1}\right)\left(\sum_{z_{2}\in Z^{n_{2}}}P\left[u_{2}+b\left(z_{1}\right)+L_{22}^{-1}z_{2}\in \lambda C_{0}^{n_{2}}\right]\right) \end{array}$$

with $F_{\lambda }\left(z_{1}\right)=\prod_{i=1}^{n_{1}}p_{\lambda ,\sigma_{i|I}}\left(z_{1}\right)$. Application of Lemma 1 to the $z_{2}$ summation, thereby following similar steps as shown in (16) and recognizing that the $b(z_{1})$ contribute as phase shifts, the result is obtained.

## References

[1] Strang G., Borre K. (1997). Linear Algebra, Geodesy, and GPS.

[2] Misra P., Enge P. (2006). Global Positioning System: Signals, Measurements and Performance.

[3] Hofmann-Wellenhof B., Lichtenegger H., Wasle E. (2008). GNSS: Global Navigation Satellite Systems. GPS, GLONASS, Galileo and More.

[4] Leick A., Rapoport L., Tatarnikov D. (2015). GPS Satellite Surveying.

[5] Morton Y., van Diggelen F., Spilker J., Parkinson B., Lo S., Gao G. (2020). Position, Navigation, and Timing Technologies in the 21st Century: Integrated Satellite Navigation, Sensor Systems, and Civil Applications.

[6] Teunissen P.J.G. (2003). Integer aperture GNSS ambiguity resolution. Artif. Satell..

[7] Euler H.J., Schaffrin B. (1990). On a measure for the discernibility between different ambiguity solutions in the static-kinematic GPS-mode. Kinematic Syst. Geod. Surv. Remote Sens..

[8] Tiberius C.C.J.M., de Jonge P.J. Fast positioning with the LAMBDA method. Proceedings of the 4th International Symposium on Differential Satellite Navigation Systems DSNS’95.

[9] Wang J., Stewart M.P., Tsakiri M. (1998). A discrimination test procedure for ambiguity resolution on-the-fly. J. Geod..

[10] Verhagen S. (2005). On the reliability of integer ambiguity resolution. Navigation.

[11] Hou Y., Verhagen S., Wu J. (2016). An efficient implementation of fixed failure-rate ratio test for GNSS ambiguity resolution. Sensors.

[12] Zhao J., Huang P., Wang Y., Sheng C., Wang L., Yu B. (2026). Model-driven generalized fixed failure rate difference test threshold determination method for GNSS integer ambiguity validation. Measurement.

[13] Teunissen P.J.G. (2005). Integer aperture bootstrapping: A new GNSS ambiguity estimator with controllable fail-rate. J. Geod..

[14] Green G.N., King M., Humphreys T. Data-driven generalized integer aperture bootstrapping for real-time high integrity applications. Proceedings of the 2016 IEEE/ION Position, Location and Navigation Symposium (PLANS).

[15] Green G.N., Humphreys T.E. (2019). Data-driven generalized integer aperture bootstrapping for high-integrity positioning. IEEE Trans. Aerosp. Electron. Syst..

[16] Green G.N., Humphreys T.E. (2019). Position-domain integrity analysis for generalized integer aperture bootstrapping. IEEE Trans. Aerosp. Electron. Syst..

[17] Zhao J., Huang P., Yu B., Wang L., Wang Y., Sheng C., Yi Q., Yang J. (2024). Optimized Integer Aperture Bootstrapping for High-Integrity CDGNSS Applications. Remote Sens..

[18] Zhao J., Huang P., Wang Y. (2026). Performance-based position domain integrity analysis for integer aperture bootstrapping estimator. Adv. Space Res..

[19] Teunissen P.J.G. (2003). Theory of Integer Equivariant Estimation with Application to GNSS. J. Geod..

[20] Teunissen P.J.G. (2026). Fourier Ambiguity Resolution for Carrier-Phase GNSS. Appli. Sci..

[21] De Jonge P.J., Tiberius C.C.J.M. (1996). The LAMBDA Method for Integer Ambiguity Estimation: Implementation Aspects.

[22] Massarweh L., Verhagen S., Teunissen P.J.G. (2025). New LAMBDA toolbox for mixed-integer models: Estimation and evaluation. GPS Solut..

[23] Verhagen S. (2005). The GNSS Integer Ambiguities: Estimation and Validation.

[24] Wei M., Schwarz K.P. Fast ambiguity resolution using an integer nonlinear programming method. Proceedings of the 8th International Technical Meeting of the Satellite Division of the Institute of Navigation (ION GPS 1995).

[25] Han S., Rizos C. (1996). Validation and rejection criteria for integer least-squares estimation. Surv. Rev..

[26] Champeney D.C. (1987). A Handbook of Fourier Theorems.

[27] Osborne A.R. (2010). Nonlinear Ocean Waves and the Inverse Scattering Transform.

[28] Cabane S., Huang S., Simons G. (1981). On the theory of elliptically contoured distributions. J. Multivar. Anal..

[29] Teunissen P.J.G. (2020). Best integer equivariant estimation for elliptically contoured distributions. J. Geod..

[30] Arnold S.F. (1990). Mathematical Statistics.

[31] Stark H., Woods J.W. (1986). Probability, Random Processes, and Estimation Theory for Engineers.

[32] Teunissen P.J.G., de Jonge P.J., Tiberius C.C.J.M. The volume of the GPS ambiguity search space and its relevance for integer ambiguity resolution. Proceedings of the ION GPS-96 (The 9th International Technical Meeting of the Satellite Division of The Institute of Navigation).

[33] Khodabandeh A., Zaminpardaz S., Nadarajah N. (2021). A study on multi-GNSS phase-only positioning. Meas. Sci. Technol..

[34] Paziewski J., Fortunato M., Mazzoni A., Odolinski R. (2021). An analysis of multi-GNSS observations tracked by recent Android smartphones and smartphone-only relative positioning results. Measurement.

[35] Yong C.Z., Odolinski R., Zaminpardaz S., Moore M., Rubinov E., Er J., Denham M. (2021). Instantaneous, Dual-Frequency, Multi-GNSS Precise RTK Positioning Using Google Pixel 4 and Samsung Galaxy S20 Smartphones for Zero and Short Baselines. Sensors.

[36] Khodabandeh A. (2022). Bias-bounded estimation of ambiguity: A method for radio interferometric positioning. IEEE Trans. Signal Process..

[37] Mohamadi A., Nahavandchi H., Khodabandeh A. (2025). Phase-Only positioning in urban environments: Assessing its potential for mass-market GNSS receivers. J. Spat. Sci..

[38] Khalife J., Kassas Z.Z.M. (2023). Performance-driven design of carrier phase differential navigation frameworks with megaconstellation LEO satellites. IEEE Trans. Aerosp. Electron. Syst..

[39] Stock W., Schwarz R.T., Hofmann C.A., Knopp A. (2024). Survey On Opportunistic PNT With Signals From LEO Communication Satellites. IEEE Commun. Surv. Tutor..

[40] Tolstov G.P., Silverman R.A. (1976). Fourier Series.

